# Quantitative relationship between activity effects and interfacial characteristics of environmental fine particles

**DOI:** 10.1093/nsr/nwaf161

**Published:** 2025-04-24

**Authors:** Xinwen Ou, Han Song, Jing Zhang, Zhang Lin

**Affiliations:** Chinese National Engineering Research Center for Control & Treatment of Heavy Metal Pollution, School of Metallurgy and Environment, Central South University, China; Hong Kong Branch of Chinese National Engineering Research Center for Tissue Restoration and Reconstruction, Department of Chemistry, The Hong Kong University of Science and Technology, China; Chinese National Engineering Research Center for Control & Treatment of Heavy Metal Pollution, School of Metallurgy and Environment, Central South University, China; National Engineering Laboratory for VOCs Pollution Control Materials & Technology, Research Center for Environmental Material and Pollution Control Technology, University of Chinese Academy of Sciences, China; Chinese National Engineering Research Center for Control & Treatment of Heavy Metal Pollution, School of Metallurgy and Environment, Central South University, China

## Abstract

A multi-factor coupling theoretical framework for interfacial activity is proposed, quantitatively uncovering the synergistic effects of particle size and specific surface energy on fine particle interfacial behavior, while offering a precise design basis for the controllable separation and stabilization of pollutants in fine particles.

Fine particles, typically characterized by at least one dimension ranging from nanometers to several micrometers, exhibit high interfacial activity and strong interactions with surrounding environmental media. They are widely found in the environment and represent an intermediate state between solutions and bulk materials. These particles possess prominent interfaces with dynamic and evolving properties [1]. Specifically, surface atoms tend to migrate into solution (dissolution), while solute atoms exhibit a propensity to attach to the interface (adsorption). These interfacial behaviors play a significant role in pollutant occurrence, species transformation, and migration, as well as in various geochemical processes of pollutants [2,3]. However, previous studies have largely focused on qualitative descriptions or simplified quantitative analyses, failing to comprehensively predict the behavior of atoms at interfaces under the influence of intrinsic interfacial factors, such as specific surface energy (surface tension) and specific surface area.

Existing theoretical models, such as the classical Gibbs–Thomson equation [4,5] and the Langmuir adsorption model [6], provide foundational insights but face notable limitations in describing the complex interfacial behaviors of fine particles. The Gibbs–Thomson equation relates particle size to solubility but does not account for the regulatory effects of adsorption on dissolution or the role of changes in specific surface energy, such as those introduced by

surface defects. Similarly, the Langmuir adsorption model describes the relationship between adsorption capacity and adsorption strength but neglects the influence of particle size and surface defects. Neither model systematically accounts for the complex interplay between specific surface energy, specific surface area, and interfacial atomic migration. These limitations underscore the necessity of a multi-factor coupling theoretical framework for interfacial activity, offering deeper insights into the interfacial behavior of fine particles.

From a thermodynamic perspective, the Gibbs free energy of fine particles (${G_{{\mathrm{nano}}}} = {G_{{\mathrm{bulk}}}} + \gamma {A_{\mathrm{s}}}$) is determined by two key interfacial factors: specific surface energy (*γ*) and specific surface area (*A*_s_). Specific surface energy reflects the instability of surface atoms due to their lower coordination numbers, weaker bonding interactions, and less ordered arrangements, while specific surface area quantifies the geometric properties of the particles (e.g. size). Furthermore, the presence of defects further enhances the specific surface energy and surface reactivity [7]. Together, these factors govern the interfacial activity of fine particles (Fig. S1), making them more chemically active than bulk materials, enabling stronger interactions with environmental pollutants. By using spherical particles as a representative model, this study systematically explores the quantitative relationship among particle size, specific surface energy, and interfacial activity (Fig. 1a).

**Figure 1.**
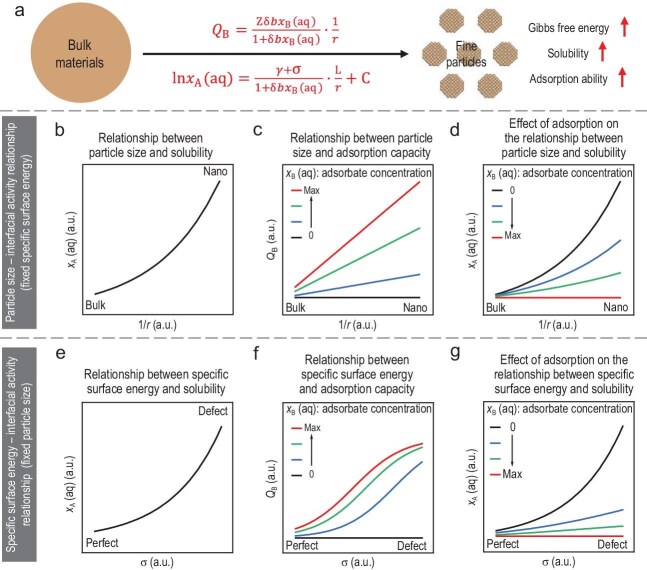
(a) Chemical activity differences between bulk materials and fine particles. (b) The relationship between particle size and solubility. (c) The relationship between particle size and adsorption capacity. (d) The relationship among particle size, adsorbate concentration, and solubility. (e) The relationship between specific surface energy and solubility. (f) The relationship between specific surface energy and adsorption capacity. (g) The relationship among specific surface energy, adsorbate concentration, and solubility.

The classical Gibbs–Thomson equation describes how solubility increases with decreasing particle size due to additional pressure induced by specific surface energy. According to the dissolution equilibrium condition (see details in Note S1), the solubility dependence on particle size at constant specific surface energy (*γ*) is expressed as:


(1) \begin{eqnarray*}
{\mathrm{ln}}{x_{\mathrm{A}}}\!\left( {{\mathrm{aq}}} \right) = \frac{{L\gamma }}{r} + C,
\end{eqnarray*}

where *x*_A_(aq) is the ratio of the concentration of dissolved A to the standard concentration, *r* is the particle radius, $L = \frac{{2{M_{\mathrm{A}}}}}{{RT\rho }}$ (*M*_A_: molar mass of particle substance A; *R*: ideal gas constant; *T*: Temperature; *ρ*: particle density). This equation indicates that solubility increases exponentially as particle size decreases (Fig. 1b), and it has been used to explain the relationship between solubility and size in some simple systems (Fig. S2). However, the classical Gibbs–Thomson equation does not account for adsorption effects or changes in specific surface energy in complex environmental systems, limiting its applicability to real environmental scenarios. This limitation highlights the need for an extended theoretical model that incorporates the interplay among particle size, specific surface energy, adsorption, and solubility.

On the other hand, the Langmuir adsorption model primarily describes the relationship between adsorption capacity and adsorption strength but does not explicitly reflect the role of specific surface area [6]. It is worth noting that the maximum adsorption capacity in the Langmuir model represents the number of adsorption sites and is thus associated with the contribution of specific surface area (particle size). Therefore, the Langmuir adsorption model can be extended by incorporating the relationship between maximum adsorption capacity and particle size (see details in Note S2). For a spherical particle, the dependence of adsorption on particle size at constant specific surface energy is expressed as:


(2) \begin{eqnarray*}
{Q_{\mathrm{B}}} = \frac{{Zb{x_{\mathrm{B}}}\!\left( {{\mathrm{aq}}} \right)}}{{1 + b{x_{\mathrm{B}}}\!\left( {{\mathrm{aq}}} \right)}}\cdot\frac{1}{r},
\end{eqnarray*}

where *Q*_B_ is the adsorption capacity, *b* is the adsorption constant, *x*_B_(aq) is the concentration of adsorbate (B) in solution, and $Z = \frac{{3k}}{\rho }$ (*k*: unit-area maximum adsorption capacity). According to this equation, adsorption capacity is inversely proportional to particle radius, meaning smaller particles exhibit higher adsorption capacities due to their larger specific surface area (Fig. 1c and Fig. S3).

As described above, both solubility and adsorption are influenced by interfacial properties, suggesting a coupled relationship between solubility and adsorption capacity in real environmental systems. Although experiments have shown that adsorption can reduce particle solubility [8,9], the quantitative relationship remains unclear. Considering that adsorption reduces the specific surface energy of particles at the solid-liquid interface, the corresponding additional pressure on fine particles also decreases. Consequently, the solubility increment caused by additional pressure is similarly affected. Here, we assume that the sites occupied by the adsorbates on the surface are no longer considered part of the solid-liquid interface and thus contribute zero to the specific surface energy of the particles. Given the relationship among additional pressure (Δ*P*), specific surface area (*A*_S_), and specific surface energy (*γ*) (${\mathrm{\Delta }}P{\mathrm{d}}{V_{\mathrm{A}}} = \gamma {\mathrm{d}}{A_{\mathrm{s}}}$), we can infer the decrease in the equivalent specific surface energy based on the reduction in effective specific surface area. Based on this (see details in Note S3), we derived the following relationship among solubility, adsorption, and particle size:


(3) \begin{eqnarray*}
{\mathrm{ln}}{x_{\mathrm{A}}}\!\left( {{\mathrm{aq}}} \right) = \frac{{L\gamma }}{{1 + b{x_{\mathrm{B}}}\!\left( {{\mathrm{aq}}} \right)}}\cdot\frac{1}{r} + C.
\end{eqnarray*}

At a constant particle size, solubility decreases with increasing adsorbate concentration, indicating that adsorption suppresses particle dissolution (Fig. 1d), which aligns with the experimental results (Fig. S4). However, as particle size decreases, both adsorption and dissolution are enhanced due to increased surface Gibbs free energy. Notably, the rate of increase in solubility with decreasing particle size is mitigated by adsorption effects. Consequently, the concentration of adsorbates in the environment is also an important factor influencing the solubility, while also explaining the enhanced colloidal stability of nanoparticles by organic ligands. It should be noted that this is an idealized model, and its application limitations are discussed in detail in Note S3.

Besides particle size, the specific surface energy also significantly affects solubility and adsorption. Surface defects, modeled as an additional specific surface energy (*σ*), enhance dissolution by increasing the thermodynamic driving force. For defective particles, the specific surface energy is expressed as $\gamma_{\rm defect} = \gamma + \sigma $. To obtain solubility dependence on specific surface energy at a constant particle size (see details in Note S4), we extend the Gibbs–Thomson equation as:


(4) \begin{eqnarray*}
{\mathrm{ln}}{x_{\mathrm{A}}}\!\left( {{\mathrm{aq}}} \right) = \frac{{L{\mathrm{(}}\gamma {\mathrm{ + }}\sigma {\mathrm{)}}}}{r} + C.
\end{eqnarray*}

This equation reveals that solubility increases exponentially with increasing specific surface energy (Fig. 1e), highlighting the role of surface defects in enhancing particle dissolution. It should be noted that, in addition to defect regulation, the additional specific surface energy is influenced by a variety of physicochemical factors, including doping, crystal structure, pH, ionic strength, and dissolved organic matter. Therefore, this equation can explain the effect of changes in specific surface energy, caused by the structural characteristics of the particles and environmental factors on solubility. For instance, the specific surface energy and solubility of SiO₂ both increase significantly with rising pH [10].

The adsorption capacity of defective particles can also be derived by extending the Langmuir adsorption model. For defective surfaces, the adsorption constant *b* is modified to account for defect-induced additional specific surface energy, expressed as ${b_{{\mathrm{defect}}}} = b{e^{\frac{{j\sigma {A_{\mathrm{s}}}}}{{RT}}}}$, where *j* is a proportionality factor of adsorption strength. By incorporating this modification into the Langmuir adsorption equation (see details in Note S5), the adsorption capacity of defective particles can be described as a function of specific surface energy and particle size:


(5) \begin{eqnarray*}
{Q_{\mathrm{B}}} = \frac{{Z\delta b{x_B}\!\left( {{\mathrm{aq}}} \right)}}{{1 + \delta b{x_B}\!\left( {{\mathrm{aq}}} \right)}}\cdot\frac{1}{r},
\end{eqnarray*}

where $\delta = {e^{\frac{{j\sigma {A_{\mathrm{s}}}}}{{RT}}}}$. This equation, referred to as the **Extended Interfacial Activity**  **Equation****(1)**, provides a comprehensive and in-depth understanding of the adsorption behavior of adsorbates at interfaces (Fig. 1f). Here, it is considered that the increase in specific surface energy is achieved by increasing the defect density, which consequently enhances the adsorption capacity. This is also consistent with previous experimental studies demonstrating the enhancement of heavy metal adsorption by surface defects (Fig. S5). The equation demonstrates that the adsorption capacity of fine particles is not solely determined by their larger specific surface area but is further amplified by the increase in specific surface energy.

To comprehensively account for the combined effects of particle size and specific surface energy on the activity behaviors of adsorption and dissolution, we further extended the Gibbs–Thomson equation (see details in Note S6) as:


(6) \begin{eqnarray*}
{\mathrm{ln}}{x_{\mathrm{A}}}\!\left( {{\mathrm{aq}}} \right) = \frac{{\gamma + {\mathrm{\sigma }}}}{{1 + \delta b{x_{\mathrm{B}}}\!\left( {{\mathrm{aq}}} \right)}}\cdot\frac{{\mathrm{L}}}{r} + C.
\end{eqnarray*}

This equation, termed the **Extended Interfacial Activity**  **Equation**** (2)**, provides a comprehensive framework that integrates the relationships among particle size, specific surface energy, adsorption, and dissolution, significantly advancing existing models of fine particle activity. Notably, the equation demonstrates that adsorption moderates the increase in solubility caused by a rise in specific surface energy (Fig. 1g). By simultaneously accounting for the effects of specific surface energy and particle size, it highlights their combined influence on the dissolution behavior of fine particles. This multi-factor coupling model overcomes the limitations of the classical models, offering deeper insight into the complex interplay among adsorption, dissolution, and interfacial properties.

This study establishes a unified theoretical framework that extends the classical Gibbs–Thomson equation and Langmuir adsorption model to comprehensively describe the quantitative relationship among particle size, specific surface energy, solubility, and adsorption capacity. The classical Gibbs–Thomson equation, which defines the relationship between particle size and solubility, can be regarded as a special case of the **Extended Interfacial Activity**  **Equation**** (2)** when the specific surface energy is constant, and the concentration of adsorbates is zero. The proposed multi-factor coupling theoretical framework for interfacial activity is broadly applicable to a wide range of fine particles and their surrounding media, offering insights into the mechanisms governing the behavior of fine particles in complex environments. In particular, it sheds light on the activity regulation of fine particles and their roles in pollutant separation and stabilization, thus establishing a theoretical foundation for precisely controlling their interfacial behavior at the atomic scale (Fig. S6). Given the widespread presence of fine particles in inorganic solid waste, regulating their interfacial behavior is not only crucial for achieving efficient heavy metal separation but also for ensuring their long-term stabilization.

## Abbreviations

aq: aqueous phase; R: ideal gas constant; T: Temperature; ${x_{\mathrm{A}}}( {{\mathrm{aq}}} )$: the ratio of the concentration of dissolved A to the standard concentration; *r*: particle radius; γ: specific surface energy; M: molar mass; ρ: particle density; A: particle substance A; B: adsorbate substance B; ${Q_{\mathrm{B}}}$: adsorption capacity; *b*: adsorption constant; *k*: unit-area maximum adsorption capacity; ${A_{\mathrm{s}}}$: specific surface area; ${x_{\mathrm{B}}}( {{\mathrm{aq}}} )$: concentration of adsorbate in solution; σ: additional specific surface energy contributed by defects; *j*: proportionality factor of adsorption strength.

## Supplementary Material

nwaf161_Supplemental_File

## References

[1] Banfield JF, Zhang H. Rev Mineral Geochem 2001; 44: 1–58. 10.2138/rmg.2001.44.01

[2] Huang X, Auffan M, Eckelman MJ et al. Nat Rev Earth Environ 2024; 5: 572–87. 10.1038/s43017-024-00567-5

[3] Hodges BC, Cates EL, Kim J-H. Nat Nanotechnol 2018; 13: 642–50. 10.1038/s41565-018-0216-x 30082806

[4] Gibbs JW . Am J Sci 1878; s3-16: 441–58. 10.2475/ajs.s3-16.96.441

[5] Thomson W . The London, Edinburgh, and Dublin Philosophical Magazine and Journal of Science 1871; 42: 448–52. 10.1080/14786447108640606

[6] Langmuir I . J Am Chem Soc 1918; 40: 1361–403. 10.1021/ja02242a004

[7] Hummer DR, Kubicki JD, Kent PRC et al. J Phys Chem C 2009; 113: 4240–5. 10.1021/jp811332w

[8] Johnson SB, Yoon TH, Kocar BD et al. Langmuir 2004; 20: 4996–5006. 10.1021/la036288y 15984260

[9] Cui W, Zhang X, Pearce CI et al. Environ Sci Technol 2020; 54: 6375–84. 10.1021/acs.est.9b07881 32298589

[10] Rimer JD, Trofymluk O, Navrotsky A et al. Chem Mater 2007; 19: 4189–97. 10.1021/cm070708d

