# Supplementary material for: Quantitative relationship between activity effects and interfacial characteristics of environmental fine particles
Source: Natl Sci Rev. 2025 Apr 24;12(6):nwaf161. doi: 10.1093/nsr/nwaf161 (PMC12104974; doi:10.1093/nsr/nwaf161)
Supplement: nwaf161_Supplemental_File [file nwaf161_supplemental_file.pdf]

Supplementary Information

Quantitative relationship between activity effects and interfacial characteristics of environmental fine particles

Xinwen Ou[1,2], Han Song[1], Jing Zhang[3] & Zhang Lin[1,*]

[1]Chinese National Engineering Research Center for Control & Treatment of Heavy Metal Pollution, School of Metallurgy and Environment, Central South University, Changsha, Hunan 410083, China;

[2]Hong Kong Branch of Chinese National Engineering Research Center for Tissue Restoration and Reconstruction, Department of Chemistry, The Hong Kong University of Science and Technology, Clear Water Bay, Kowloon, Hong Kong 999077, China;

[3]National Engineering Laboratory for VOCs Pollution Control Materials & Technology, Research Center for Environmental Material and Pollution Control Technology, University of Chinese Academy of Sciences, Beijing 101408, China

[*]**Corresponding author.** E-mail: zhang_lin@csu.edu.cn

Supplemental Information

Supplementary Notes 1 to 8

Figures S1 to S6

Supplementary Note 1. Dependence of Solubility on Particle Size at Constant Specific Surface Energy

The classical Gibbs-Thomson equation has been used to describe the relationship between particle solubility and size [1-6]. Specifically, as particle size decreases, solubility increases exponentially due to the additional pressure induced by specific surface energy (surface tension). For spherical particles, the additional pressure is expressed as $\Delta P = \frac{2\gamma}{r}$, where γ and r are the specific surface energy (at solid-liquid interface) and particle radius. As particle size decreases, the additional pressure and chemical potential increase, leading to enhanced dissolution. According to previous studies [7, 8], the classical Gibbs-Thomson equation is derived as follows.

At equilibrium, the chemical potential of a particle in the solid phase equals that in the solution:

$$\mu_{\mathrm{A}}(\mathrm{s}, r, T, P+\Delta P) = \mu_{\mathrm{A}}(\mathrm{aq}, T, P, x_{\mathrm{A}}(\mathrm{aq})) \qquad \text{(S1A)}$$

Differentiating both sides yields:

$$\mathrm{d}\mu_{\mathrm{A}}(\mathrm{s}, r, T, P+\Delta P) = \mathrm{d}\mu_{\mathrm{A}}(\mathrm{aq}, T, P, x_{\mathrm{A}}(\mathrm{aq})) \qquad \text{(S1B)}$$

$$-S_{\mathrm{A}}(\mathrm{s})\mathrm{d}T + V_{\mathrm{A}}(\mathrm{s})\mathrm{d}(P + \Delta P) = -S_{\mathrm{A}}(\mathrm{aq})\mathrm{d}T + V_{\mathrm{A}}(\mathrm{aq})\mathrm{d}P + RT\mathrm{dln}x_{\mathrm{A}}(\mathrm{aq}) \qquad \text{(S1C)}$$

For constant external pressure and temperature, the equation simplifies to:

$$V_{\mathrm{A}}(\mathrm{s})\mathrm{d}(\Delta P) = RT\mathrm{dln}x_{\mathrm{A}}(\mathrm{aq}) \qquad \text{(S1D)}$$

$$\frac{M_A}{\rho}\mathrm{d}\frac{2\gamma}{r} = RT\mathrm{dln}x_{\mathrm{A}}(\mathrm{aq}) \qquad \text{(S1E)}$$

Considering only changes in particle size, it yields:

$$\frac{2M_A\gamma}{\rho}\mathrm{d}\frac{1}{r} = RT\mathrm{dln}x_{\mathrm{A}}(\mathrm{aq}) \qquad \text{(S1F)}$$

Integrating both sides:

$$\int RT\mathrm{dln}x_{\mathrm{A}}(\mathrm{aq}) = \int \frac{2M_A\gamma}{\rho}\mathrm{d}\frac{1}{r} + \mathrm{C} \qquad \text{(S1G)}$$

$$\mathrm{ln}x_{\mathrm{A}}(\mathrm{aq}) = \frac{2M_A\gamma}{RT\rho r} + \mathrm{C} \qquad \text{(S1H)}$$

For simplification, we define $\mathrm{L} = \frac{2M_A}{RT\rho}$, yielding:

$$\mathrm{ln}x_{\mathrm{A}}(\mathrm{aq}) = \frac{\mathrm{L}\gamma}{r} + \mathrm{C} \qquad \text{(S1I)}$$

The unit of L is m^3/J, which can also be expressed as m^2/N or Pa^{-1}, consistent with the dimension of compressibility. Compressibility characterizes the degree of particle deformation under unit pressure, highlighting its relationship with the additional pressure generated by specific surface energy. The integration constant C corresponds to the natural logarithm of the ratio of bulk material solubility to the standard concentration, which approximates that of an infinitely large, intact surface. Herein, the terms are defined as follows: μ: chemical potential; s: solid phase; aq: aqueous phase; R: ideal gas constant; T: Temperature; P: pressure; ΔP: additional pressure; A: particle substance A; $x_{\mathrm{A}}(\mathrm{aq})$: the ratio

of the concentration of dissolved A to the standard concentration; S: molar entropy; V: molar volume; M: molar mass; ρ: particle density.

Supplementary Note 2. Dependence of Adsorption on Particle Size at Constant Specific Surface Energy

The classical Langmuir adsorption model primarily describes the relationship between adsorption capacity and adsorption strength but does not consider the influence of specific surface area [9]. Analysis reveals that the contribution of specific surface area is embedded within the maximum adsorption capacity of the Langmuir model. Here, we extend the Langmuir adsorption equation to integrate the relationship between specific surface area and maximum adsorption capacity and thus determine the relationship between adsorption capacity and particle size.

Under a given external pressure and temperature, the system reaches adsorption equilibrium:

$$Q_B = \frac{Q_{\mathrm{B}}^0 b x_{\mathrm{B}}(\mathrm{aq})}{1+b x_{\mathrm{B}}(\mathrm{aq})} \qquad \text{(S2A)}$$

Where b, Q_{B} and Q_B^0 are the adsorption constant, adsorption capacity, and the maximum adsorption capacity, respectively. For a specific material, the adsorption constant b, also known as the adsorption affinity, is considered to be related to the binding strength between the adsorbate and the adsorption site. The adsorption constant is theoretically related to the specific surface energy and is influenced by various physicochemical factors, including defects, doping, crystal structure, pH, ionic strength, and dissolved organic matter (DOM). Here, a simplified model assuming constant specific surface energy is considered. A detailed discussion on how specific surface energy affects the adsorption constant is provided in Supplementary Note 5. The maximum adsorption capacity, Q_B^0, represents the number of adsorption sites and is proportional to the specific surface area (A_s). For spherical particles, $A_s = \frac{3}{\rho r}$, where ρ and r is the density and radius of the particle. Substituting $Q_B^0 = \mathrm{k} \cdot A_s$, where k is the unit-area maximum adsorption capacity.

$$Q_B^0 = \mathrm{k} \cdot A_s = \frac{3\mathrm{k}}{\rho r} \qquad \text{(S2B)}$$

$$Q_B = \frac{3\mathrm{k} b x_{\mathrm{B}}(\mathrm{aq})}{\rho(1+b x_{\mathrm{B}}(\mathrm{aq}))} \cdot \frac{1}{r} \qquad \text{(S2C)}$$

For simplicity, we define $\mathrm{Z} = \frac{3\mathrm{k}}{\rho}$, yielding:

$$Q_{\mathrm{B}} = \frac{Z b x_B(\mathrm{aq})}{1+b x_B(\mathrm{aq})} \cdot \frac{1}{r} \qquad \text{(S2D)}$$

The unit of Z is m·kg/kg, and its dimension is consistent with the particle radius (m). It quantifies the sensitivity of adsorption performance to variations in particle size. Note that the unit of Q_{B} is kg/kg, representing the mass of adsorbate adsorbed per unit mass of adsorbent.

Herein, the terms are defined as follows: b: adsorption constant; B: adsorbate substance B; $x_{\mathrm{B}}(\mathrm{aq})$: concentration of adsorbate in solution.

It should be noted that the Langmuir adsorption model, although widely used for adsorption studies in aqueous solutions, was originally derived for solid-gas interfaces. In aqueous environments, the model approximates the processes such as surface dehydration and adsorbate desolvation by treating them as part of the solvent effect. Despite these simplifications, the Langmuir model remains a reliable and practical tool for studying adsorption performance [10-13] and predicting adsorbate removal efficiency or the required amount of adsorbent in aqueous solutions [14].

Supplementary Note 3. Relationship Among Solubility, Adsorption, and Particle Size

Equations (S1I) and (S2D) indicate that both solubility and adsorption depend on the interfacial properties of particles, suggesting a coupled relationship between solubility and adsorption capacity in real environmental systems. Although previous experiments have demonstrated that adsorption can reduce particle solubility [15-18], precise quantitative relationship has yet to be clarified. To address this, we further derive the influence of adsorption capacity on the relationship between solubility and particle size. The occupation of adsorbates can be treated as a reduction in the specific surface energy that is required for the surface work ($\gamma \mathrm{d}A_s$) of fine particle formation. Considering the source of additional pressure ($\Delta P \mathrm{d}V_{\mathrm{A}} = \gamma \mathrm{d}A_s$), this is equivalent to a decrease in additional pressure, thereby reducing the solubility of fine particles. Since the sites occupied by adsorbates are no longer considered part of the solid-liquid interface, their contribution to the specific surface energy of the solid-liquid interface is regarded as zero. Based on the assumption of a single-layer adsorption model, the relationship between the equivalent specific surface area and adsorption amount can be established. The adsorption amount is Q_B, and the surface area occupied by the adsorbate is $\frac{Q_B}{Q_B^0} \cdot A_s$, and thus the unoccupied surface area is $\frac{Q_B^0 - Q_B}{Q_B^0} \cdot A_s$. Due to the relationship among additional pressure, specific surface energy, and specific surface area, the equivalent specific surface energy of the particle can be expressed as:

$$\gamma_{\mathrm{equivalent}} = \frac{Q_{\mathrm{B}}^0 - Q_{\mathrm{B}}}{Q_{\mathrm{B}}^0} \cdot \gamma = \frac{1}{1 + b x_{\mathrm{B}}(\mathrm{aq})} \cdot \gamma \qquad \text{(S3A)}$$

Substituting equivalent specific surface energy into Equation (S1H):

$$\ln x_{\mathrm{A}}(\mathrm{aq}) = \frac{2M_A \gamma}{RT\rho r} \cdot \frac{1}{1 + b x_B(\mathrm{aq})} + \mathrm{C} \qquad \text{(S3B)}$$

For simplicity, we rewrite this as:

$$\ln x_{\mathrm{A}}(\mathrm{aq}) = \frac{\mathrm{L}\gamma}{1 + b x_{\mathrm{B}}(\mathrm{aq})} \cdot \frac{1}{r} + \mathrm{C} \qquad \text{(S3C)}$$

It should be noted that here this model is based on the assumption that there is no interaction between the dissolved particle substance A and free adsorbate substance B in solution. Under

this assumption, the concentration of dissolved ions is not influenced by processes such as coordination, chelation, redox reactions, or other interactions that could disrupt the dissolution equilibrium. These processes could significantly alter dissolution behavior and lead to deviations from the predictions of our model. For example, some organic acids enhance mineral dissolution by bringing surface-coordinated atoms into solution or coordinating with atoms in the solution phase, a phenomenon known as ligand-promoted dissolution [19-21].

At the same time, the model assumes a simplified scenario that the particle surface is homogeneous, meaning all sites are equivalent, and all sites function as both adsorption and dissolution sites. Consequently, the reduction in specific surface energy caused by adsorption is directly treated as equivalent to the reduction in solubility. However, in cases where not all dissolution sites serve as adsorption sites, or if the particle surface is heterogeneous (e.g., certain crystal faces contribute more to adsorption while others dominate dissolution), the actual solubility may deviate from the prediction of the equation.

Supplementary Note 4. Dependence of Solubility on Specific Surface Energy at Constant Particle Size

Specific surface energy is another critical interfacial property influencing the activity effects of fine particles. Changes in specific surface energy caused by surface defects (e.g., vacancies, kinks, steps, and grain boundaries) can significantly impact dissolution behavior. To explore this, we extend the Gibbs-Thomson equation to account for changes in specific surface energy. Here, γ is the specific surface energy of a perfect (defect-free) solid particle in solution, and σ represents the additional specific surface energy contributed by defects. For a defective particle (at constant particle size) in solution, the specific surface energy is $\gamma_{\mathrm{defect}} = \gamma + \sigma$. According to Equation (S1E):

$$\frac{2M_A}{\rho r}\mathrm{d}\gamma_{\mathrm{defect}} = RT\mathrm{d}\ln x_{\mathrm{A}}(\mathrm{aq}) \qquad \text{(S4A)}$$

$$\ln x_{\mathrm{A}}(\mathrm{aq}) = \frac{2M_{\mathrm{A}}\gamma_{\mathrm{defect}}}{RT\rho r} + \mathrm{C} \qquad \text{(S4B)}$$

Substituting the specific surface energy of defective particle, it can be obtained that:

$$\ln x_{\mathrm{A}}(\mathrm{aq}) = \frac{2M_A(\gamma+\sigma)}{RT\rho r} + \mathrm{C} \qquad \text{(S4C)}$$

For simplicity, we rewrite this as:

$$\ln x_{\mathrm{A}}(\mathrm{aq}) = \frac{\mathrm{L}(\gamma+\sigma)}{r} + \mathrm{C} \qquad \text{(S4D)}$$

It should be noted that, in addition to defect regulation, the additional specific surface energy is influenced by a variety of physicochemical factors, including doping, crystal structure, pH, ionic strength, and dissolved organic matter (DOM). Therefore, this equation can explain the

effect of changes in specific surface energy, caused by the structural characteristics of the particles and environmental factors, on solubility.

Supplementary Note 5. Adsorption Capacity as a Function of Specific Surface Energy and Particle Size

The relationship between adsorption capacity and specific surface energy can be derived by extending the Langmuir adsorption equation. The adsorption constant *b* in the Langmuir model represents the equilibrium constant of the adsorption reaction and is directly related to the Gibbs free energy of adsorption (ΔG). When the adsorption constant, *b*, is normalized by b_0 (a unit cancellation constant) to make it dimensionless, the Gibbs free energy of the adsorption on the perfect surface can be expressed as:

$$\Delta G = -RT\ln\frac{b}{b_0} \qquad \text{(S5A)}$$

For defective particles in solution, the formation energy of defects increases the specific surface energy, which can be expressed as $\gamma_{\mathrm{defect}} = \gamma + \sigma$. The increase in specific surface energy, i.e. σ, contributes to the surface Gibbs free energy by σA_s. The increase in the surface Gibbs free energy enhances the adsorption strength (since adsorption energy is negative, the adsorption strength is set as the negative adsorption energy for convenience). Assuming the increase in adsorption strength is proportional to the increase in the surface Gibbs free energy, the proportionality factor is denoted as *j*. The difference in the Gibbs free energy of adsorption reactions between the perfect surface and the defective surface (ΔΔG), which reflects the difference in their adsorption energies (with the entropy contribution assumed to be identical for both perfect and defective particles), can be expressed as:

$$\Delta\Delta G = \mathrm{j}\sigma A_s \qquad \text{(S5B)}$$

$$\Delta\Delta G = -RT\ln\frac{b}{b_0} + RT\ln\frac{b_{\mathrm{defect}}}{b_0} = RT\ln\frac{b_{\mathrm{defect}}}{b} \qquad \text{(S5C)}$$

$$RT\ln\frac{b_{\mathrm{defect}}}{b} = \mathrm{j}\sigma A_s \qquad \text{(S5D)}$$

Thus, the adsorption constant for defective particles becomes:

$$b_{\mathrm{defect}} = be^{\frac{\mathrm{j}\sigma A_s}{RT}} \qquad \text{(S5E)}$$

Substituting this into Equation (S2C):

$$Q_B = \frac{3\mathrm{k}be^{\frac{\mathrm{j}\sigma A_s}{RT}}x_{\mathrm{B}}(\mathrm{aq})}{\rho(1+be^{\frac{\mathrm{j}\sigma A_s}{RT}}x_{\mathrm{B}}(\mathrm{aq}))}\cdot\frac{1}{r} \qquad \text{(S5F)}$$

For simplicity, we define $\delta = e^{\frac{\mathrm{j}\sigma A_s}{RT}}$, yielding:

$$Q_{\mathrm{B}} = \frac{Z\delta b x_B(\mathrm{aq})}{1+\delta b x_B(\mathrm{aq})}\cdot\frac{1}{r} \qquad \text{(S5G)}$$

We refer to this equation as the **Extended Interfacial Activity Equation (1)**.

It should be noted that, in addition to enhancing adsorption strength, surface defects may also influence the density of adsorption sites. Both mechanisms contribute to adsorption capacity, although their effects are distinct. As illustrated in previous studies [22, 23], if defects primarily enhance adsorption strength (adsorption affinity), the adsorption capacity for low-concentration adsorbates will increase significantly. On the other hand, if defects primarily increase the density of adsorption sites (maximum adsorption capacity), the overall adsorption capacity will increase, but the effect may be less pronounced at lower concentrations. Therefore, to determine the enhancement effect of defects on adsorption capacity, adsorption amounts can be calculated using area normalization method [24-26].

Supplementary Note 6. Relationship Among Solubility, Adsorption, Specific Surface Energy, and Particle Size

The adsorption of adsorbates onto the particle surface can be regarded as an effective reduction in the specific surface energy. Consequently, the effect of adsorption is treated as an equivalent decrease in the specific surface energy of the defective particles. The equivalent specific surface energy can be expressed as:

$$(\gamma+\sigma)_{\text{equivalent}}=\frac{Q_{\mathrm{B}}^{0}-Q_{\mathrm{B}}}{Q_{\mathrm{B}}^{0}}\cdot(\gamma+\sigma)=\frac{\gamma+\sigma}{1+be^{\frac{\mathrm{j}\sigma A_{\mathrm{S}}}{\mathrm{RT}}}x_{\mathrm{B}}(\mathrm{aq})} \qquad \text{(S6A)}$$

By incorporating the effects of adsorption on particle solubility (Equation (S4C)), it yields:

$$\ln x_{\mathrm{A}}(\mathrm{aq})=\frac{2M_{\mathrm{A}}}{RT\rho r}\cdot\frac{\gamma+\sigma}{1+be^{\frac{\mathrm{j}\sigma A_{\mathrm{S}}}{\mathrm{RT}}}x_{B}(\mathrm{aq})}+\mathrm{C} \qquad \text{(S6B)}$$

For simplicity, we rewrite:

$$\ln x_{\mathrm{A}}(\mathrm{aq})=\frac{\gamma+\sigma}{1+\delta b x_{\mathrm{B}}(\mathrm{aq})}\cdot\frac{\mathrm{L}}{r}+\mathrm{C} \qquad \text{(S6C)}$$

We refer to this equation as the **Extended Interfacial Activity Equation (2)**. It is similarly derived based on an equivalence model, as in the derivation of Equation (S3C), and thus shares the same application limitations.

Supplementary Note 7. Environmental Case Studies and Validation

The interfacial behavior and activity effects of environmental fine particles are determined by the Gibbs free energy of the system. As shown in Fig. S1a, the Gibbs free energy of alumina increases linearly with increasing specific surface area [27]. Similarly, the potential energy per unit of TiO_2 increases as particle size decreases (Fig. S1b), highlighting the critical role of

specific surface area in determining the interfacial properties of fine particles [28]. On the other hand, the small size and surface defects of fine particles lead to more disordered atomic arrangements and lower stability, resulting in higher specific surface energy. For example, Hummer et al. [29] demonstrated through XRD measurements and DFT calculations that the specific surface energy of TiO_2 increases as particle size decreases, with this increase primarily attributed to the defects associated with edges and corners (Figs S1c-d). Similarly, Zheng et al. found that CdS nanocrystals exhibit high intrinsic defect concentrations, which decrease as the crystals grow larger [30]. Additionally, electroplating sludge contains a large number of amorphous nanoparticles that feature abundant point defects, dislocations, and planar defects [31]. These findings emphasize the importance of considering particle size and surface defects when studying the activity effects of environmental fine particles.

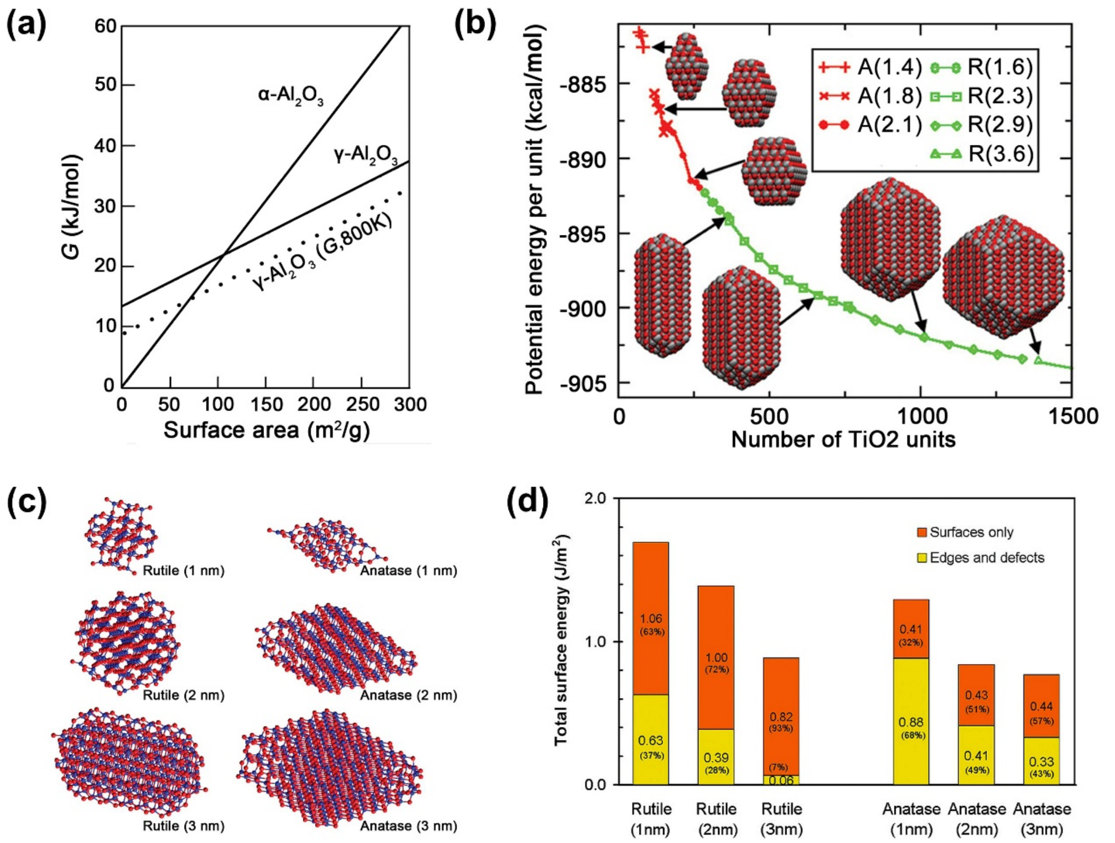

Figure S1. **(a)** Calculated Gibbs free energy of alumina polymorphs relative to coarse corundum based on surface energies obtained through molecular dynamics simulations. From Ref. [27]. Reprinted with permission from AAAS. **(b)** Potential energy of a series of (quasi-)Wulff shapes of anatase (A, red symbols) and rutile (R, green symbols) as a function of the number of TiO_2 units. Reprinted with permission from Ref. [28]. Copyright 2012 American Chemical Society. **(c)** Energy minimized model TiO_2 nanoparticles. **(d)** Total DFT calculated surface energies of model 1, 2, and 3 nm anatase and rutile particles. Red represents the contribution from the sum of the particle's constituent crystallographic surfaces. Yellow represents the contribution from edges and defects. Reprinted with permission from Ref. [29].

Copyright 2009 American Chemical Society.

The Gibbs-Thomson equation shows that solubility increases exponentially as particle size decreases (Fig. S2a). Mudunkotuwa et al. [5] studied the solubility of ZnO particles of different sizes, and their fitting results were consistent with the Gibbs-Thomson equation (Fig. S2b). Sugimoto et al. also observed similar size-dependent solubilities in these silver halide particles [4]. Additionally, particle dissolution kinetics are also closely related to size, with smaller particles dissolving significantly faster than larger ones. Zhu et al. found that the dissolution rate of fine hematite particles far exceeded that of larger particles [1]. Echigo et al. also found that the initial dissolution rate of 7 nm hematite particles was significantly higher than that of 30 nm hematite particles [3]. The high solubility of fine particles strongly influences pollutant migration, transformation, and ecological toxicity. For instance, atmospheric particulate matter exhibits strong dissolution abilities during aging, releasing large amounts of heavy metals that induce oxidative stress and cytotoxicity [32, 33]. On the other hand, regulating particle size offers an effective means of controlling dissolution behavior, presenting a practical strategy for pollution remediation.

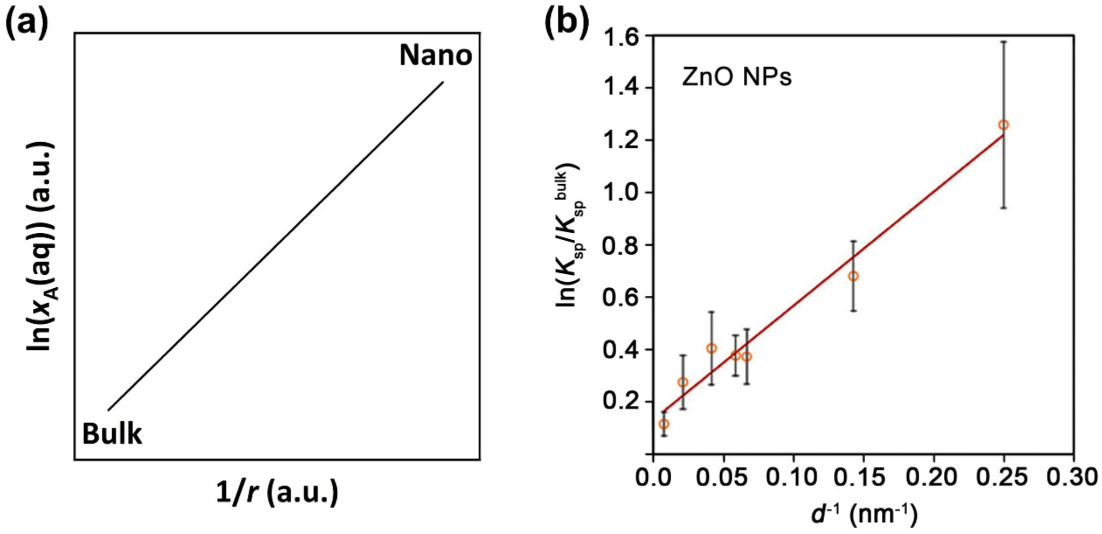

Figure S2. (**a**) The relationship between solubility and particle size. (**b**) Size-dependent dissolution of ZnO nanoparticles. Note that the solubility is represented as a natural logarithm function. Reprinted with permission from Ref. [5]. Copyright 2012 American Chemical Society.

As shown in Fig. S3a, adsorption capacity is inversely proportional to particle size, highlighting the advantages of nanoparticles in pollutant adsorption and water treatment. For example, Xu et al. [34] found that the adsorption capacity of $Mg(OH)_2$ for Sb(III) increased as particle size decreased (Fig. S3b). In addition, nanoscale zero-valent iron (nZVI) exhibits significantly higher adsorption capacity for As(V) compared to larger ZVI particles, making it

highly effective for water treatment and environmental remediation [35]. Similarly, atmospheric particulate matter, as natural fine particles, exhibits high adsorption capacities for pollutants such as polycyclic aromatic hydrocarbons (PAHs) and heavy metals due to its large specific surface area [36]. More importantly, the adsorption behavior of particles also affects their solubility. As shown in Fig. S4a, increasing adsorbate concentrations result in higher adsorption capacities and lower solubilities. Johnson et al. [16] demonstrated that the solubility of aluminum oxide decreases as the maleate concentration in solution increases, which is attributed to the strong adsorption of maleate on the surface (Fig. S4b). Cui et al. [18] discovered an armoring effect caused by adsorbed Cr(III) on boehmite nanoparticles, which significantly reduces the number of dissolution-active sites on particle surfaces and, consequently, greatly decreases their solubility. Kowalczyk et al. [17] also found that surfactants strongly adsorb onto calcite surfaces, reducing the dissolution rate of calcite by a factor of 5–6 after coating with surfactants.

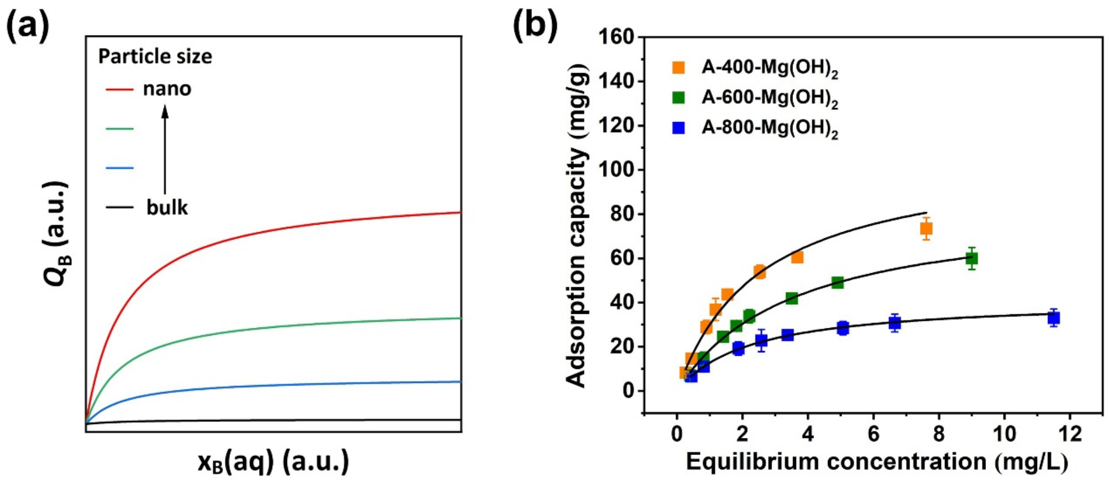

Figure S3. (**a**) Adsorption capacity as a function of adsorbate concentration and particle size. **(b)** Adsorption isotherms of Sb(III) on $Mg(OH)_2$ with varying particle sizes. Reprinted from Ref. [34], with permission from Elsevier.

The suppression effect of surface adsorption of fine particles on their dissolution can be utilized to regulate the growth mechanism of nanoparticles by controlling their surface adsorption state. For example, in a highly concentrated NaOH solution (4M), ZnS nanoparticles exhibit significantly reduced dissolution rates due to the strong adsorption of NaOH on their surfaces, which keeps the nanoparticles in an undersaturated condition. As a result, during the early stages of hydrothermal coarsening, the classical Ostwald ripening (OR) growth mechanism—typically requiring a saturated solution—is suppressed, giving way to a novel multistep oriented attachment (OA) growth mechanism (Fig. S4d) [37]. On the other hand, the adsorption effect of fine particles can be used to enhance their thermodynamic stability. In the synthesis of nanomaterials, organic ligands are often employed to improve the

thermodynamic stability of the colloidal semiconductor nanocrystals [38]. Yuan et al. discovered that the adsorption of mercaptoethanol can stabilize 13 nm MnS nanoparticles, whereas particles without ligand adsorption are only stable at much larger sizes (~200 nm) [39]. Similarly, Huang et al. found that the adsorption of mercaptopropionic acid on CdTe quantum dots can effectively control their particle size [40]. Interestingly, Lin et al. [41] found that ZnS in highly concentrated NaOH solutions (17 M) are even more stable in their nanoscale phase than in their bulk phase, owing to the strong adsorption of NaOH on the surface of ZnS nanoparticles, which results in a negative specific surface energy. Calvin et al. [42] also observed that some prototypical semiconductor nanocrystals (InP, ZnS, CdS, PbS) exhibit a negative specific surface energy upon the adsorption of oleic acid on their surfaces. This is due to the strong bonding interactions between surface atoms and adsorbed oleic acid, which render surface atoms thermodynamically even more stable than those within the crystal interior.

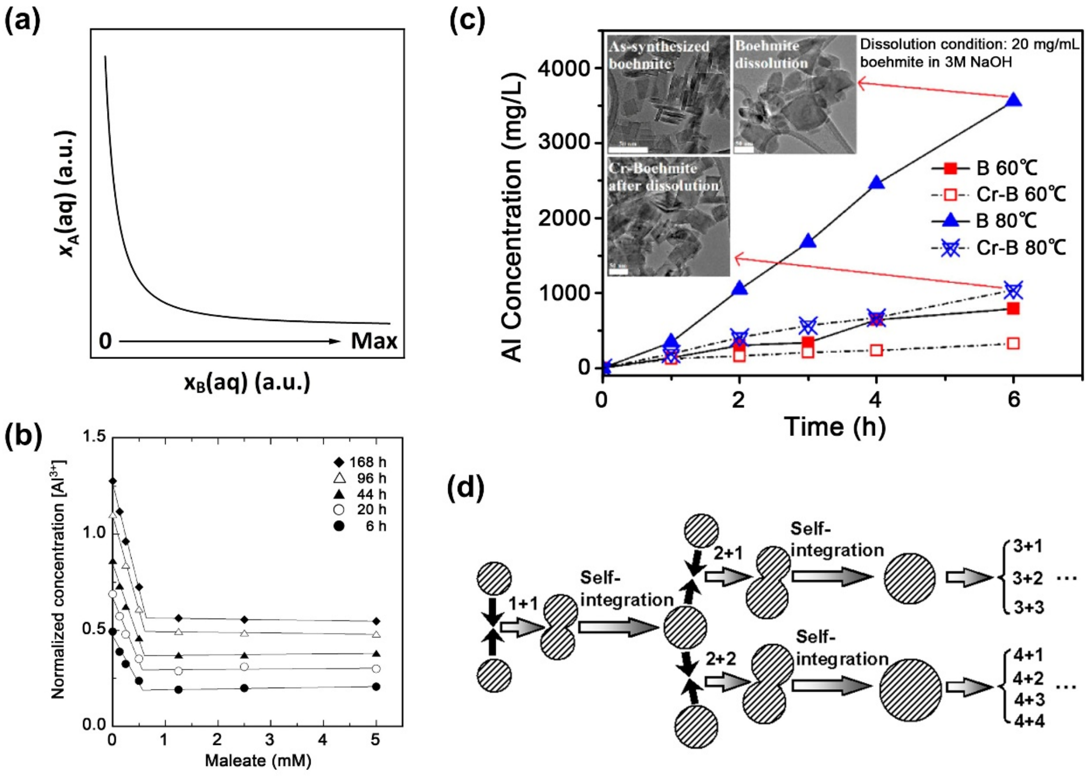

Figure S4. **(a)** The relationship between solubility and adsorbate concentration. (**b**) Concentration of Al^{3+} dissolved from corundum as a function of maleate concentration and time. Reprinted with permission from Ref. [16]. Copyright 2004 American Chemical Society. (**c**) Time-dependence of boehmite and Cr(III)-adsorbed boehmite dissolution. Reprinted with permission from Ref. [18]. Copyright 2020 American Chemical Society. (**d**) Scheme of the OA-based growth of nanoparticles. Reprinted with permission from Ref. [37]. Copyright 2006 American Chemical Society.

As shown in Fig. S5a, defects on particle surfaces significantly increase specific surface energy, thereby enhancing adsorption capacity. For example, Ou et al. demonstrated that defect-rich $Mg(OH)_2$ exhibited much higher adsorption capacities for arsenate and chromate than defect-poor $Mg(OH)_2$ [24]. Similarly, Zhan et al. found that introducing oxygen vacancies into BiOCl materials enhanced the adsorption ability for Cr(VI) [25]. Liu et al. [26] reported that the adsorption of arsenate on hematite surfaces increased with the concentration of oxygen vacancy defects (Figs S5b-d). These findings suggest that defect engineering can be used to enhance the adsorption capacity of fine particles for heavy metals and other pollutants.

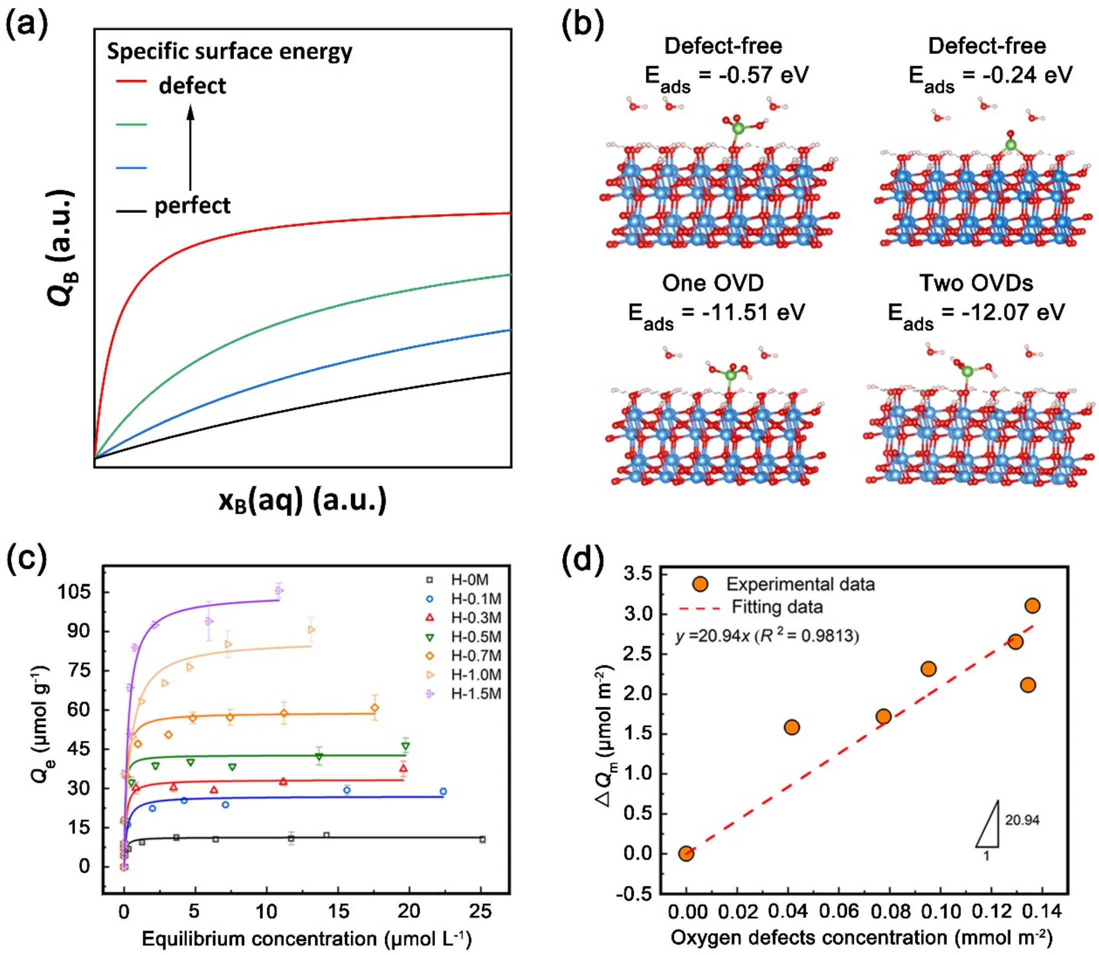

Figure S5. (**a**) Adsorption capacity as a function of adsorbate concentration and specific surface energy. (**b**) Optimized structure of inner-sphere As(V) absorbed on the surface of the hematite slab with the absence and presence of oxygen vacancy defects. (**c**) Adsorption isotherm curves of different hematite samples with respect to As(V) at pH 6.0. (**d**) Relationship between the oxygen vacancy concentration and arsenic adsorption capacity. Reprinted with permission from Ref. [26]. Copyright 2023 American Chemical Society.

Supplementary Note 8. Environmental Implications

Besides dissolved forms, fine particles serve as the primary medium for heavy metals in the environment. For example, Guo et al. found that 20-45% of arsenic (As) in groundwater exists in colloidal fine particles [43]. Zhou et al. reported that fine particle aggregates are the primary carriers of heavy metals, such as Cr, Cu, Zn, Ni, As, Cd, and Pb, in contaminated paddy soils [44]. Lin and co-workers discovered that brownmillerite, hydrogarnet, and amorphous phase [45], as well as brucite[46] and gypsum [47], all on the micro- and nanoscale, are important host phases of Cr(VI) in chromium-containing hazardous waste. Notably, the activity of these metastable fine particles can easily change, which in turn affects the immobilization of heavy metals. For instance, Lei et al. observed that the dissolution rate of Cr(III) significantly increases as the particle size of $(Cr_x, Fe_{1-x})(OH)_3$ decreases. Furthermore, under UV light, $(Cr_x, Fe_{1-x})(OH)_3$ itself acts as a catalyst to accelerate the oxidation of Cr(III), ultimately leading to a substantial increase in the generation rate of Cr(VI) in solution [48]. During the transformation of amorphous fine particles, such as ferrihydrite, into crystalline hematite and goethite, adsorbed Cr(VI) [49] and Cd(II) [50], can be re-released into the solution. This is because the growth of fine particles results in reduced defects and specific surface area, thereby lowering surface reactivity and facilitating the desorption of heavy metals. Therefore, in practical engineering applications, the rapid growth of fine particles can reduce the specific surface area and specific surface energy, thereby passivating interfacial activity and facilitating the separation of heavy metals from particles (Fig. S6a). For example, Lin and co-workers realized deep removal of Cr(VI) from hazardous waste by controlling the phase transformation processes of fine particles, such as magnesium hydrate [46] and gypsum [47]. Meanwhile, to immobilize the free heavy metals in the environment, the creation of surface defects in fine particles can be regulated to increase surface energy, thereby enhancing surface activity (Fig. S6b). For instance, the presence of surface defects can significantly improve the adsorption and oxidation activity of manganese oxide to As(III) [51]. For fine particles formed by heavy metal salt aggregation or heavy metal doping, promoting crystal growth into bulk phases can reduce the specific surface energy and solubility, thereby achieving long-term immobilization of heavy metals (Fig. S6c). For example, Wang et al. used a sintering process to promote the crystallization and growth of heavy metals (Cu, Pb, Zn, Cd) in iron tailings, reducing their leaching rate and achieving effective immobilization [52]. In other words, the ability of fine particles to immobilize heavy metals during structural transformation is primarily controlled by both specific surface energy and specific surface area. By directionally regulating the specific surface energy and specific surface area of fine particles, both long-term immobilization and deep extraction of heavy metals can be achieved.

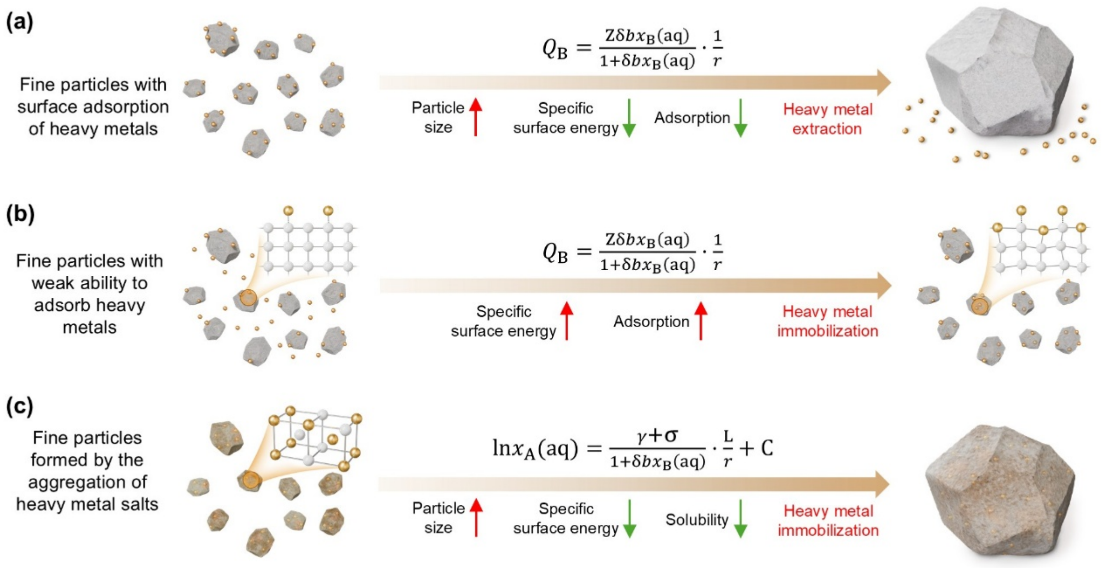

Figure S6. (**a**) Growth of fine particles reduces interfacial activity, promoting the desorption and extraction of heavy metals. (**b**) Defect creation enhances the adsorption and immobilization of heavy metals by fine particles. (**c**) The growth of fine particles formed by the aggregation of heavy metal salts reduces solubility and promotes heavy metal immobilization.

REFERENCES

1. Zhu, G, Legg, BA, Sassi, M *et al.* Crystal dissolution by particle detachment. *Nat Commun* 2023; **14**: 6300.
2. Liu, J, Aruguete, DM, Murayama, M *et al.* Influence of size and aggregation on the reactivity of an environmentally and industrially relevant nanomaterial (pbs). *Environ Sci Technol* 2009; **43**: 8178-83.
3. Echigo, T, Aruguete, DM, Murayama, M *et al.* Influence of size, morphology, surface structure, and aggregation state on reductive dissolution of hematite nanoparticles with ascorbic acid. *Geochim Cosmochim Acta* 2012; **90**: 149-62.
4. Sugimoto, T, Shiba, F. A new approach to interfacial energy. 3. Formulation of the absolute value of the solid−liquid interfacial energy and experimental collation to silver halide systems. *J Phys Chem B* 1999; **103**: 3607-15.
5. Mudunkotuwa, IA, Rupasinghe, T, Wu, CM *et al.* Dissolution of zno nanoparticles at circumneutral ph: A study of size effects in the presence and absence of citric acid. *Langmuir* 2012; **28**: 396-403.
6. Liu, L, Long, Z, Shi, K *et al.* A general crystallization picture of quantum dots: The underlying physical chemistry. *CCS Chemistry* 2025; **7**: 926-49.
7. Gibbs, JW. On the equilibrium of heterogeneous substances. *Am J Sci* 1878; **3**: 441-58.
8. Thomson, W. On the equilibrium of vapour at a curved surface of liquid. *Philos Mag* 1871; **42**: 448-52.
9. Langmuir, I. The adsorption of gases on plane surfaces of glass, mica and platinum. *J Am Chem Soc* 1918; **40**: 1361-403.
10. Alsbaiee, A, Smith, BJ, Xiao, L *et al.* Rapid removal of organic micropollutants from water by a porous beta-cyclodextrin polymer. *Nature* 2016; **529**: 190-4.
11. Ghosal, PS, Gupta, AK. Determination of thermodynamic parameters from langmuir

isotherm constant-revisited. *J Mol Liq* 2017; **225**: 137-46.
12. Guo, X, Wang, J. Comparison of linearization methods for modeling the langmuir adsorption isotherm. *J Mol Liq* 2019; **296**: 111850.
13. Basar, CA. Applicability of the various adsorption models of three dyes adsorption onto activated carbon prepared waste apricot. *J Hazard Mater* 2006; **135**: 232-41.
14. Chung, H-K, Kim, W-H, Park, J *et al.* Application of langmuir and freundlich isotherms to predict adsorbate removal efficiency or required amount of adsorbent. *Journal of Industrial and Engineering Chemistry* 2015; **28**: 241-6.
15. Liu, J, Sonshine, DA, Shervani, S *et al.* Controlled release of biologically active silver from nanosilver surfaces. *ACS Nano* 2010; **4**: 6903-13.
16. Johnson, SB, Yoon, TH, Kocar, BD *et al.* Adsorption of organic matter at mineral/water interfaces. 2. Outer-sphere adsorption of maleate and implications for dissolution processes. *Langmuir* 2004; **20**: 4996-5006.
17. Kowalczyk, B, Bishop, KJ, Lagzi, I *et al.* Charged nanoparticles as supramolecular surfactants for controlling the growth and stability of microcrystals. *Nat Mater* 2012; **11**: 227-32.
18. Cui, W, Zhang, X, Pearce, CI *et al.* Effect of cr(iii) adsorption on the dissolution of boehmite nanoparticles in caustic solution. *Environ Sci Technol* 2020; **54**: 6375-84.
19. Ludwig, C, Devidal, J-L, Casey, WH. The effect of different functional groups on the ligand-promoted dissolution of nio and other oxide minerals. *Geochim Cosmochim Acta* 1996; **60**: 213-24.
20. Perry, TDt, Duckworth, OW, Kendall, TA *et al.* Chelating ligand alters the microscopic mechanism of mineral dissolution. *J Am Chem Soc* 2005; **127**: 5744-5.
21. Wang, Z, Schenkeveld, WD, Kraemer, SM *et al.* Synergistic effect of reductive and ligand-promoted dissolution of goethite. *Environ Sci Technol* 2015; **49**: 7236-44.
22. Cao, Q, Huang, F, Zhuang, Z *et al.* A study of the potential application of nano-mg(oh)2 in adsorbing low concentrations of uranyl tricarbonate from water. *Nanoscale* 2012; **4**: 2423-30.
23. Tian, C, Zhao, J, Ou, X *et al.* Enhanced adsorption of p-arsanilic acid from water by amine-modified uio-67 as examined using extended x-ray absorption fine structure, x-ray photoelectron spectroscopy, and density functional theory calculations. *Environ Sci Technol* 2018; **52**: 3466-75.
24. Ou, X, Liu, X, Liu, W *et al.* Surface defects enhance the adsorption affinity and selectivity of mg(oh)2 towards as(v) and cr(vi) oxyanions: A combined theoretical and experimental study. *Environ Sci Nano* 2018; **5**: 2570-8.
25. Zhan, G, Li, J, Hu, Y *et al.* The surface hydroxyl and oxygen vacancy dependent cr(vi) adsorption performance of biocl. *Environ Sci Nano* 2020; **7**: 1454-63.
26. Liu, J, Xiang, Y, Chen, Y *et al.* Quantitative contribution of oxygen vacancy defects to arsenate immobilization on hematite. *Environ Sci Technol* 2023; **57**: 12453-64.
27. McHale, JM, Auroux, A, Perrotta, AJ *et al.* Surface energies and thermodynamic phase stability in nanocrystalline aluminas. *Science* 1997; **277**: 788-91.
28. Zhou, Y, Fichthorn, KA. Microscopic view of nucleation in the anatase-to-rutile transformation. *J Phys Chem C* 2012; **116**: 8314-21.
29. Hummer, DR, Kubicki, JD, Kent, PRC *et al.* Origin of nanoscale phase stability reversals in titanium oxide polymorphs. *J Phys Chem C* 2009; **113**: 4240-5.
30. Zheng, J, Huang, F, Yin, S *et al.* Correlation between the photoluminescence and oriented attachment growth mechanism of cds quantum dots. *J Am Chem Soc* 2010; **132**: 9528-30.
31. Zhuang, Z, Xu, X, Wang, Y *et al.* Treatment of nanowaste via fast crystal growth: With recycling of nano-sno2 from electroplating sludge as a study case. *J Hazard Mater* 2012; **211-212**: 414-9.
32. Song, X, Wu, D, Chen, X *et al.* Toxic potencies of particulate matter from typical industrial plants mediated with acidity via metal dissolution. *Environ Sci Technol* 2024; **58**: 6736-43.
33. Sadiq, NW, Venter, C, Mohammed, W *et al.* Dissolution of selected trace elements

from simulated atmospheric aerosol aging and human exposure of mineral dust and coal fly ash. *ACS ES&T Air* 2023; **1**: 5-15.
34. Xu, S, Zhong, Z, Liu, W *et al.* Removal of sb(iii) from wastewater by magnesium oxide and the related mechanisms. *Environ Res* 2020; **186**: 109489.
35. Wu, C, Tu, J, Liu, W *et al.* The double influence mechanism of ph on arsenic removal by nano zero valent iron: Electrostatic interactions and the corrosion of fe0. *Environ Sci Nano* 2017; **4**: 1544-52.
36. Chen, Y, Zhang, L, Wu, Y *et al.* Cytotoxicity and epithelial barrier toxicity of fine particles from residential biomass pellet burning. *Environ Sci Technol* 2024; **58**: 17786-96.
37. Zhang, J, Lin, Z, Lan, Y *et al.* A multistep oriented attachment kinetics: Coarsening of zns nanoparticle in concentrated naoh. *J Am Chem Soc* 2006; **128**: 12981-7.
38. Calvin, JJ, Brewer, AS, Alivisatos, AP. The role of organic ligand shell structures in colloidal nanocrystal synthesis. *Nat Synth* 2022; **1**: 127-37.
39. Yuan, Y, Yan, X, Wang, Y *et al.* Interface effects on the phase transition of mns nanocrystal. *Surf Interfaces* 2021; **23**: 101015.
40. Huang, Y, Zhuang, Z, Xue, X *et al.* Growth kinetics study revealing the role of the mpa capping ligand on adjusting the growth modes and pl properties of cdte qds. *CrystEngComm* 2014; **16**: 1547-52.
41. Lin, Z, Gilbert, B, Liu, Q *et al.* A thermodynamically stable nanophase material. *J Am Chem Soc* 2006; **128**: 6126-31.
42. Calvin, JJ, Brewer, AS, Crook, MF *et al.* Observation of negative surface and interface energies of quantum dots. *Proc Natl Acad Sci USA* 2024; **121**: e2307633121.
43. Guo, H, Zhang, B, Zhang, Y. Control of organic and iron colloids on arsenic partition and transport in high arsenic groundwaters in the hetao basin, inner mongolia. *Appl Geochem* 2011; **26**: 360-70.
44. Zhou, M, Hu, P, Wang, J *et al.* Aqueous and colloidal dynamics in size-fractionated paddy soil aggregates with multiple metal contaminants under redox alternations. *Environ Sci Technol* 2024; **58**: 18222-33.
45. Liu, Z, Zheng, J, Liu, W *et al.* Identification of the key host phases of cr in fresh chromite ore processing residue (copr). *Sci Total Environ* 2020; **703**: 135075.
46. Liu, W, Huang, F, Liao, Y *et al.* Treatment of crvi-containing mg(oh)2 nanowaste. *Angew Chem Int Ed* 2008; **120**: 5701-4.
47. Liu, W, Zheng, J, Ou, X *et al.* Effective extraction of cr(vi) from hazardous gypsum sludge via controlling the phase transformation and chromium species. *Environ Sci Technol* 2018; **52**: 13336-42.
48. Lei, D, Gou, C, Wang, C *et al.* Visible light accelerates cr (iii) release and oxidation in cr–fe chromite residues: An overlooked risk of cr (vi) reoccurrence. *Environ Sci Technol* 2022; **56**: 17674-83.
49. Liang, C, Fu, F, Tang, B. Mn-incorporated ferrihydrite for cr(vi) immobilization: Adsorption behavior and the fate of cr(vi) during aging. *J Hazard Mater* 2021; **417**: 126073.
50. Zhou, Z, Muehe, EM, Tomaszewski, EJ *et al.* Effect of natural organic matter on the fate of cadmium during microbial ferrihydrite reduction. *Environ Sci Technol* 2020; **54**: 9445-53.
51. Hou, J, Luo, J, Hu, Z *et al.* Tremendous effect of oxygen vacancy defects on the oxidation of arsenite to arsenate on cryptomelane-type manganese oxide. *Chem Eng J* 2016; **306**: 597-606.
52. Wang, G, Ning, X-a, Lu, X *et al.* Effect of sintering temperature on mineral composition and heavy metals mobility in tailings bricks. *Waste Manage* 2019; **93**: 112-21.
